# Mitigation of salinity induced negative impacts by salt tolerant plant growth promoting rhizobacteria *Bacillus flexus* in mustard (*Brassica juncea* L.)

**DOI:** 10.3389/fmicb.2025.1638366

**Published:** 2025-07-22

**Authors:** Anjali Singh, Vishal Prasad

**Affiliations:** Institute of Environment and Sustainable Development, Banaras Hindu University, Varanasi, India

**Keywords:** *Bacillus flexus*, biopriming, mustard, plant growth promoting bacteria, rhizosphere, salinity stress

## Abstract

Salinity is a major environmental stressor affecting crop productivity worldwide and a substantial portion of the agricultural ecosystem supporting cultivation of mustard (*Brassica juncea* L.) in Indian subcontinent is salinity stricken. However, plant growth promoting rhizobacteria has been noted to modulate salinity stress in plants through numerous direct and indirect mechanisms. Therefore, the present study was intended to determine the potential of a salt tolerant plant growth promoting rhizobacteria *Bacillus flexus* on alleviating the negative effects of salinity stress in mustard. The evaluation of germination percentage, growth parameters, and pigment content (chlorophyll and carotenoids) along with biochemical properties and antioxidant enzyme activities of mustard was studied by biopriming the seeds with *Bacillus flexus* both in absence and presence of salinity (100 mM NaCl) stress. The obtained results revealed a significant improvement in germination percentage and growth parameters (shoot length, root length, biomass and leaf area) of bioprimed mustard seedling both in presence and absence of salt stress. The biochemical properties such as pigment content, proline, total soluble protein, and total soluble sugar were found improved in bacterial treated seeds in comparison to control both in presence and absence of salinity stress. The percentage of electrolyte leakage and malondialdehyde (MDA) content was found decreased in bacterial treated plants under salinity induced condition as compared with non-treated plants. The antioxidant enzymes such as catalase (CAT), peroxidise (POX) and ascorbate peroxidise (APX) activities were found elevated in bacterial treated seeds in comparison to control both in presence and absence of salinity stress. The results obtained from the study revealed the protective and growth promoting abilities of *Bacillus flexus* against salinity stress. The bacterial strain used in the present study proved to be a promising candidate for improving mustard growth in soils challenged with salinity stress.

## 1 Introduction

Among various environmental stressors, salinity is one of the major and deleterious abiotic stressor which affects plant productivity substantially. Approximately 5.2 billion hectares of fertile land globally is stricken by different kinds of soil degradation problem. Out of which around 50% of this land is solitarily affected by problem of salinity (Numan et al., 2018; Singh and Prasad, 2020; Kumawat et al., 2023) which not only affects the soil fertility and plant growth but conjointly the living organisms (Bharti et al., 2016; Egamberdieva et al., 2019; AbuQamar et al., 2024).

Mustard (*Brassica juncea*) is a popular multipurpose plant belonging to family *Brassicaceae*. Its popularity in India and other countries is because of its oil content, which is edible and also has some medicinal value such as anti-inflammatory, anti-microbial and potential anti-cancer properties. The residues of plant have been used as cattle feed, biofuels and as a fertilizer (Shanker et al., 2011; Sharma et al., 2013; Ahmad et al., 2015). India, in spite of being largest producer of edible oil faces shortage to satisfy the daily requirement of its people (Ahmad et al., 2015). Mustard serves as a good model plant, because it often experiences salinity stress in arid and semiarid regions of the world (Parti et al., 2003). Salinity imposes ionic and osmotic stress that interferes with the growth, biomass yield, and physio-biochemical attributes of mustard seedlings (Parti et al., 2003; Sarma et al., 2012). The most common salinity impact on mustard plant could be a general stunting of plant growth, hefty delay and reduction in seed germination and seedlings growth characteristics (Ahmad et al., 2015). A significant portion of the agricultural ecosystem supporting cultivation of mustard in Indian subcontinent is salinity affected; therefore, it is imperative to investigate performance of mustard under salinity stress.

Plant growth promoting rhizobacteria (PGPR) has been noted to modulate salinity stress in plants through numerous direct and indirect mechanisms such as production of growth hormone indole acetic acid (IAA), solubilization of phosphorus, production of 1-aminocyclopropane-1-carboxylate (ACC) deaminase enzyme, ammonia and siderophore (Bharti et al., 2016; Bokhari et al., 2019; Arora et al., 2021). PGPR are known for their role in improving plant-water relations, hormonal signaling, ion homeostasis, antioxidant enzyme regulation and photosynthetic efficiency in plants exposed to salinity stress (Abbas et al., 2019; Gupta and Pandey, 2019; Ansari et al., 2025). These mechanisms are regulated by a complex network of signaling owing to their interaction and consequently ensuing stress alleviation (Habib et al., 2016; Ilanumaran and Smith, 2017; Kumawat et al., 2022). Under saline conditions, high level of Na^+^ not only hampers the uptake of nutrients but also causes peculiar ion toxicity (Barnawal et al., 2012; Slimani et al., 2023). The PGPR strains have the potential to protect the plants from deleterious effects of high Na^+^ concentration in saline soils and they do so by their ability to produce ACC-deaminase enzyme, stimulation of growth hormone and accumulation of osmoprotectors which protect the plants from injurious effects of the salinity stress (Abbas et al., 2019; Gupta and Pandey, 2019; Kibret et al., 2024). The ability of PGPR to produce exopolysaccharides (EPS) is also reported to limit the Na^+^ uptake in plants by their binding with it and biofilms (Yang et al., 2009; Qurashi and Sabri, 2013). The lowered availability of Na^+^ ends up in its reduced uptake thereby balancing high K^+^/Na^+^ ratio that facilitate the plant to survive better under salinity stress condition (Nadeem et al., 2014; Egamberdieva et al., 2019; Mukhtar et al., 2020; Kumawat et al., 2023). An efficient survival of rhizobacteria under saline condition is due to excessive accumulation of secondary metabolites may help in better colonization and prove beneficial in prospering strategies to promote plant growth in saline soils (Qurashi and Sabri, 2013). Several of the rhizobacterial strains such as *Bacillus, Pseudomonas, Klebsiella, Azotobacter, Enterobacter* etc. have been reported to influence the growth and development of wheat, tomatoes, peppers, beans, and lettuce grown in saline environments (Nadeem et al., 2014; Ahmad et al., 2015; Orhan, 2016; Egamberdieva et al., 2019; Kumawat et al., 2023). It has been proved from various studies that rhizobacteria from salinity affected soil with multiple PGP traits along with salt tolerance properties are more likely to endure salinity stress and would be suitable for use in sustainable agricultural practices (Mukhtar et al., 2020; Joshi et al., 2021; Kumawat et al., 2023).

Considering these facts, the present study was intended to evaluate the effectiveness of a salt tolerant plant growth promoting rhizobacterial strain *Bacillus flexus* for alleviating the negative impacts of salinity stress on mustard seedlings.

## 2 Materials and methods

### 2.1 Sampling and isolation of rhizobacteria

Soil samples were collected from the rhizosphere of mustard (*Brassica juncea*) plant from different salt affected agricultural sites of Azamgarh, Uttar Pradesh, India (26°03′N 83°13′E). For the isolation, 1 g of rhizospheric soil was taken with 10 mL of normal saline and mixed properly by shaking. Serial dilution was performed up to 10^−6^ fold dilution and 100 μL of suspension from each dilution was spread on nutrient agar (NA) media plates. Plates were incubated at 30°C for 24 h. The visible growth of bacterial colonies was selected for further tests.

### 2.2 Screening for salt tolerance

All the selected bacterial colonies were tested for their salt tolerance potential. All cultures along with DH5α (an *E. coli* strain) taken as a standard were streaked on nutrient agar medium supplemented with different concentration of NaCl salt (100 mM, 500 mM, 1 M, 1.5 M, and 2 M) making it selective medium. Based on observations of visible growth of the bacterial isolates on these plates after 24 h of incubation at 30°C they were considered positive or negative for their tolerance against salinity. A salt tolerant rhizobacterial strain *Bacillus flexus* (accession number MK968766) was used in the present study. This strain was able to grow on nutrient agar solid media amended with NaCl up to 2 molars (M).

### 2.3 Seed coating and germination experiment

**Figure 1:**
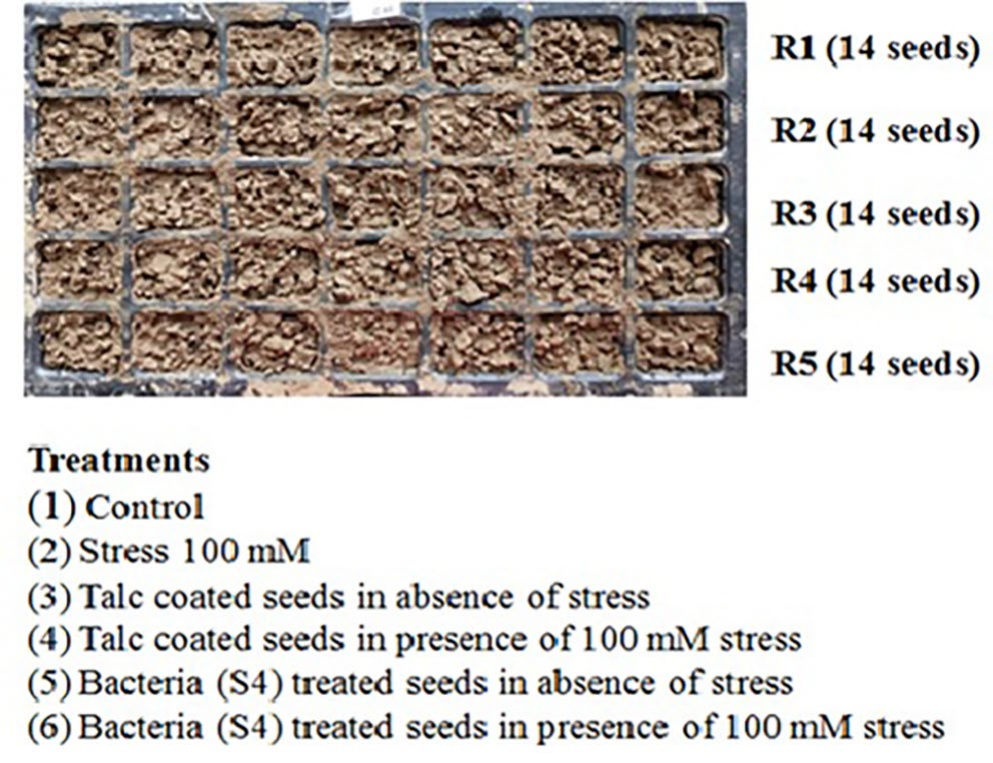
Experimental setups in germination trays.

Mustard seed (*Brassica juncea* cv. Varuna T59) were used for this study. Seeds were surface sterilized for 30 s with 70% ethyl alcohol followed by sterilization with 2% sodium hypochlorite for 2 min. Further seeds were subjected to 3 times washing with sterile distilled water. The seeds were sown in germination trays (7 columns and 5 rows, total 35 wells) with duplicate tray for each treatment and this experimental setup was kept for 21 days. Two seeds per well was sown, thus a total of 140 seeds were used for each treatment (Figure 1). Total 6 combinations of seed treatment were used in this study which was as follows;

1. Control without NaCl.
2. Control with NaCl (100 mM)
3. Talc without NaCl.
4. Talc with NaCl (100 mM)
5. Formulation of *Bacillus flexus* without NaCl.
6. Formulation of *Bacillus flexus* with NaCl (100 mM)

### 2.4 Inoculum preparation and growth condition

For making the bacterial formulation *Bacillus flexus* was grown in LB broth in a shaking incubator at 150 rpm for 18 h at 30°C. After incubation time the fully grown culture was taken and centrifuged at 3,000 rpm for 3 min. Following this the bacterial pellet was washed with sterile distilled water and re-suspended in normal saline (0.85%) solution and the culture suspension OD was maintained at A_600nm_ = 1.0. Seeds were mixed with jaggery syrup followed by coating with bacterial formulation which was prepared by mixing 1.8 × 10^7^ bacterial cells with 2 grams of talc. Talc was used as a carrier material in the bacterial formulation. The jaggery syrup was used to provide the adhesion of bacteria to the seed surface. Coated seeds were incubated overnight at 28 ± 2°C for proper adhesion of bacteria on seeds along with control and talc coated seeds. The control, talc and bacteria coated seeds were watered with distilled water in absence of salinity stress (no NaCl), while to induce artificial salinity stress in germinating mustard seedlings a solution of 100 mM NaCl (~ 10 dS/m) in distilled water was used for watering. Watering of seeds was done every alternate day.

The germination trays were kept outside the laboratory to simulate natural environmental condition. The experiment was performed from September–October 2024. The average temperature during the experiment ranged between 25.5°C–30.2°C and the relative humidity was approximately 79%.

### 2.5 Physico-chemical characteristics

The soil used in the experiment was garden soil of Institute of Environment and Sustainable Development, Banaras Hindu University, Varanasi, India with following physico-chemical characteristics as described in Table 1.

**Table 1:** Physico-chemical properties of experimental soil.

Texture	pH	EC (dS/m)	Organic Carbon (g/kg)	Total N (g/kg)	Total P (g/kg)	Total K (g/kg)
Clay loam	8.1	0.642	0.32	0.21	0.05	0.32

### 2.6 Germination and growth analysis of mustard seedlings

Germination was recorded on a daily basis for 21 days and at the end of experimental period total percentage of germination was calculated. The time (days) taken by seeds for 50% germination (T_50_) was calculated by following the formula of Farooq et al. (2018).

$T_{50}=\mathrm{ti}+[(N/2–\mathrm{ni})\;(\mathrm{ti}-\mathrm{tj})]/\mathrm{ni}–\mathrm{nj}$ .

Where, *N* denotes final number of seeds germinated, *ni* and *nj* represents total number of seeds germinated by adjacent count at time *ti* and *tj* respectively, where *ni* < *N*/2 < *nj*.

### 2.7 Estimation of seedling biomass

Mustard seedlings were collected after 21 days of germination. Seedlings were randomly selected from each treatment for the estimation of growth-related parameters. The fresh weight (g) of plants (5 plants) from each treatment was taken immediately while dry weight (g) of plants was calculated after drying the seedling in oven at 60°C for 24 h (Forti et al., 2020). The root and shoot lengths of seedlings were measured on millimetric paper. Leaf area was calculated by using following formula (Mckee, 1964).

Leaf area=Length of leaf(cm)×width of leaf(cm)×0.74 .

### 2.8 Estimation of electrolyte leakage

Electrolyte leakage (EL) was determined according to the method of Gonzalez and Gonzalez-Villar (2003). For the estimation of EL, 200 mg of fresh mustard leaves were cut into small pieces and placed into test tube, filled with 10 mL of deionized water and covered with plastic caps. Tubes were heated at a constant temperature of 32°C for 2 h in a water bath. The initial electrical conductivity (EC_1_) of the solution was recorded after 2 h by conductivity probe. Further the samples were autoclaved at 121°C for 20 min to properly kill/rupture the tissues facilitating in release of all the electrolytes. The final electrical conductivity (EC_2_) was recorded after cooling the samples at room temperature. The EL was measured using the following formula.

EL(%)=(EC1/EC2)×100 .

Where, EL represents electrolyte leakage and EC depict electrical conductivity.

### 2.9 Estimation of photosynthetic pigments

The pigments chlorophyll a, chlorophyll b, total chlorophyll and carotenoids were estimated from fresh leaves of mustard seedlings. One hundred milligrams leaf sample was placed in a test tube containing 10 mL of 80% acetone, kept for overnight in a refrigerator at 4°C. Later the sample was homogenized and centrifuged at 5,000 rpm for 7 min. The final volume of the supernatant was made up to 25 mL with 80% acetone. Absorbance of 80% acetone extracted samples were recorded at 480 nm and 510 nm for carotenoids and at 645 nm and 663 nm for chlorophyll estimation by using spectrophotometer (Thermo-Scientific, Evolution-201) (Arnon, 1949).

### 2.10 Biochemical properties and antioxidant enzymatic activity of mustard seedlings

#### 2.10.1 Proline

For measurement of proline, method of Bates et al. (1973) was followed. For this, 100 mg of leaves were homogenized with 10 mL of 3% aqueous sulphosalicylic acid and centrifuged at 10,000 rpm for 15 min at 4°C. From this 1 mL of supernatant was taken and mixed with 1 mL of glacial acetic acid and 1 mL of ninhydrin. Mixture was incubated for 1 h at 90°C. After incubation time, samples were cooled immediately in ice water and subsequently 2 mL of toluene was added and vortexed for 2 min. The upper phase (wine red color) was collected and absorbance was recorded at 520 nm by using spectrophotometer (Thermo-Scientific, Evolution-201). Proline concentration was calculated by using the proline standard curve and reported as μg/g fresh weight.

#### 2.10.2 Total soluble protein

The estimation of total protein content was done as per Bradford method (Bradford, 1976). For this 100 mg of plant material was taken with 1 mL of protein extraction buffer (10 mM of Tris HCL and EDTA (pH 8.0), 0.1 mg/mL phenylmethylsulfonyl fluoride (PMSF) and 5 mM β-mercaptoethanol) and homogenized in a chilled mortar and pestle. This solution was collected in a 1.5 mL microcentrifuge tube and centrifuged at 12,000 rpm for 20 min at 4°C. From this 100 μL of supernatant was taken in a test tube and 3 mL of Bradford reagent was added to it, mixed properly and incubated for 10 min at room temperature. After that OD was recorded at 595 nm. The standard curve was prepared by using different concentration of Bovine serum albumin (BSA) with a range of 5–100 μg protein.

#### 2.10.3 Total soluble sugar

Total soluble sugar (mg/g) was estimated by following the PSA method of Dubois et al. (1956). For the analysis of total sugar in mustard seedlings 0.5 g fresh leaves were added to 10 mL of 80% acetone and heated at 80°C for 1 h in water bath. From this solution 0.5 mL aliquot was taken and mixed with 1 mL of phenol (5%) and 5 mL of 96% H_2_SO_4_. Solution was mixed properly and incubated for 1 h at room temperature. After 1 h reaction mixture was properly mixed by shaking and absorbance was recorded at 490 nm.

#### 2.10.4 Lipid peroxidation

Lipid peroxidation (LPO) was estimated in terms of the production of malondialdehyde (MDA) due to the thiobarbituric acid (TBA) reaction (Heath and Packer, 1968). For the LPO measurement, 500 mg of fresh leaf were taken into 5 mL of ice-cold extracting solution (5% trichloroacetic acid, TCA), homogenized in a mortar and pestle and centrifuged at 10,000 rpm for 10 min. One milliliter of extract was collected and mixed with 4 mL of 0.5% thiobarbituric acid (prepared in 20% TCA), boiled for 30 min on water bath. The solution was cooled immediately in an ice bath and centrifuged at 1,000 rpm for 10 min. The absorbance of supernatant was recorded at 532 nm and 600 nm by using spectrophotometer (Thermo-Scientific, Evolution-201). MDA content was calculated in terms of its molar extinction coefficient (155 mM^−1^ cm^−1^).

$\mathrm{LPO}={\mathrm{OD}}_{532}–{\mathrm{OD}}_{600}/155\times 1,000$ .

#### 2.10.5 Catalase

Catalase (CAT) activity was estimated according to the method of Chandlee and Scandalios (1984). For this, 100 mg of fresh plant material was taken and added to 10 mL of potassium phosphate buffer (0.1 M, pH 7). Plant material was crushed properly in to phosphate buffer and centrifuged at 10,000 rpm for 15 min. From this 0.4 mL of enzyme extract was taken and mixed with 2.6 mL of potassium phosphate buffer and 0.4 mL of hydrogen peroxide (H_2_O_2_). Solution was mixed properly and incubated for 1 min at 25°C. Catalase activity was determined in terms of reduction in absorbance of H_2_O_2_ at 240 nm.

#### 2.10.6 Peroxidase

The estimation of peroxidase (POX) enzyme was done by following the method of Kar and Mishra (1976). For peroxidase analysis, 100 mg of fresh plant material was crushed with 10 mL of potassium phosphate buffer (0.1 M, pH 7) and centrifuged at 10,000 rpm for 15 min. From the solution 0.5 mL of enzyme extract was taken and mixed with 2 mL of phosphate buffer, 1 mL of pyrogallol and 1 mL of H_2_O_2_ and incubated for 5 min at 25°C. After 5 min, reaction was terminated by adding 1 mL of 200 mM H_2_O_2_. The quantity of purpurogallin produced was estimated by taking absorbance at 420 nm against blank and enzyme activity was expressed as change in U mg^−1^ protein. Here, 1 U defined as the change in the absorbance of 0.1 min^−1^ mg^−1^ protein.

#### 2.10.7 Ascorbate peroxidase

The method of Nakano and Asada (1981) was followed for the estimation of ascorbate peroxidase (APOX) activity. For this, 100 mg of fresh plant material was taken and added to 10 mL of potassium phosphate buffer (0.1 M, pH 7) containing 2 mM ascorbate. Plant material was crushed properly in to phosphate buffer and centrifuged at 10,000 rpm for 15 min. The 3 mL reaction mixture contained 50 mM phosphate buffer (pH 7.0), 0.1 mM EDTA, 0.5 mM ascorbic acid; 0.1 mM H_2_O_2_ and 0.5 mL of enzyme extract were mixed properly. The reaction was carried out for 5 min at 25°C. Absorbance was recorded at 290 nm. All the enzyme activities were expressed as unit mg^−1^ protein.

### 2.11 Statistical analysis

One way analysis of variance (ANOVA) was performed to determine the significant differences between each treatment by using the software SPSS version 20, which was followed by Duncan’s multiple range test (DMRT) to compare the means. All the values were presented as mean ± standard error of the three replicates. Means with a significance value *p* ≤ 0.05 were considered statistically different. The significant differences among all samples were indicated by different alphabetic letters.

## 3 Results

### 3.1 Growth dynamics of rhizobacteria coated mustard seedling challenged with salt stress

#### 3.1.1 Germination percentage

**Figure 2:**
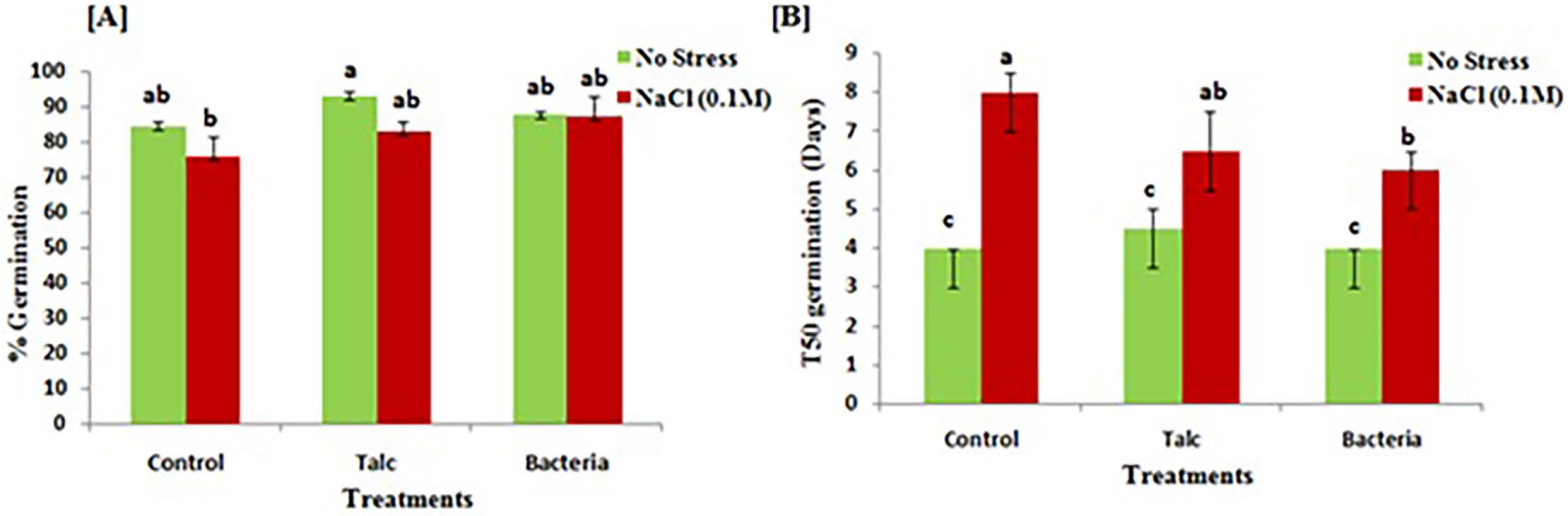
Effects of salinity stress on **(A)** germination and **(B)** time taken for 50% germination (T_50_) by mustard seeds both in presence and absence of bacterial treatment. Data presented are mean ± SE of three replicates. Different letters indicate statistically significant difference between each treatment (DMRT *p* < 0.05).

The results of seedling germination of treated and non-treated seeds are shown in Figure 2. The result of germination percentage showed that in absence of salinity stress talc coated seeds performed best (92 ± 1.43%) however, statistically no significant difference was observed in germination of seeds coated with bacterial formulation and control seeds without any salt treatment (87 ± 0.71% and 84 ± 1.23% respectively). In case of seeds having salinity exposure, minimum germination (75 ± 5.71%) was recorded in seeds which were subjected to salinity stress without any coating, while in seeds which were coated with bacteria 87 ± 5.71% germination was observed followed by talc coating (82 ± 2.85%) (Figure 2A). The time taken by seeds for all treatments to show 50% germination (T_50_) is represented in Figure 2B. There was no significant difference observed for T_50_ germination in between the treatment in absence of salinity stress. However, in presence of salt stress minimum time for 50% germination was observed for bacterial coated seeds while maximum was recorded for non-coated control seeds.

#### 3.1.2 Seedling biomass

**Figure 3:**
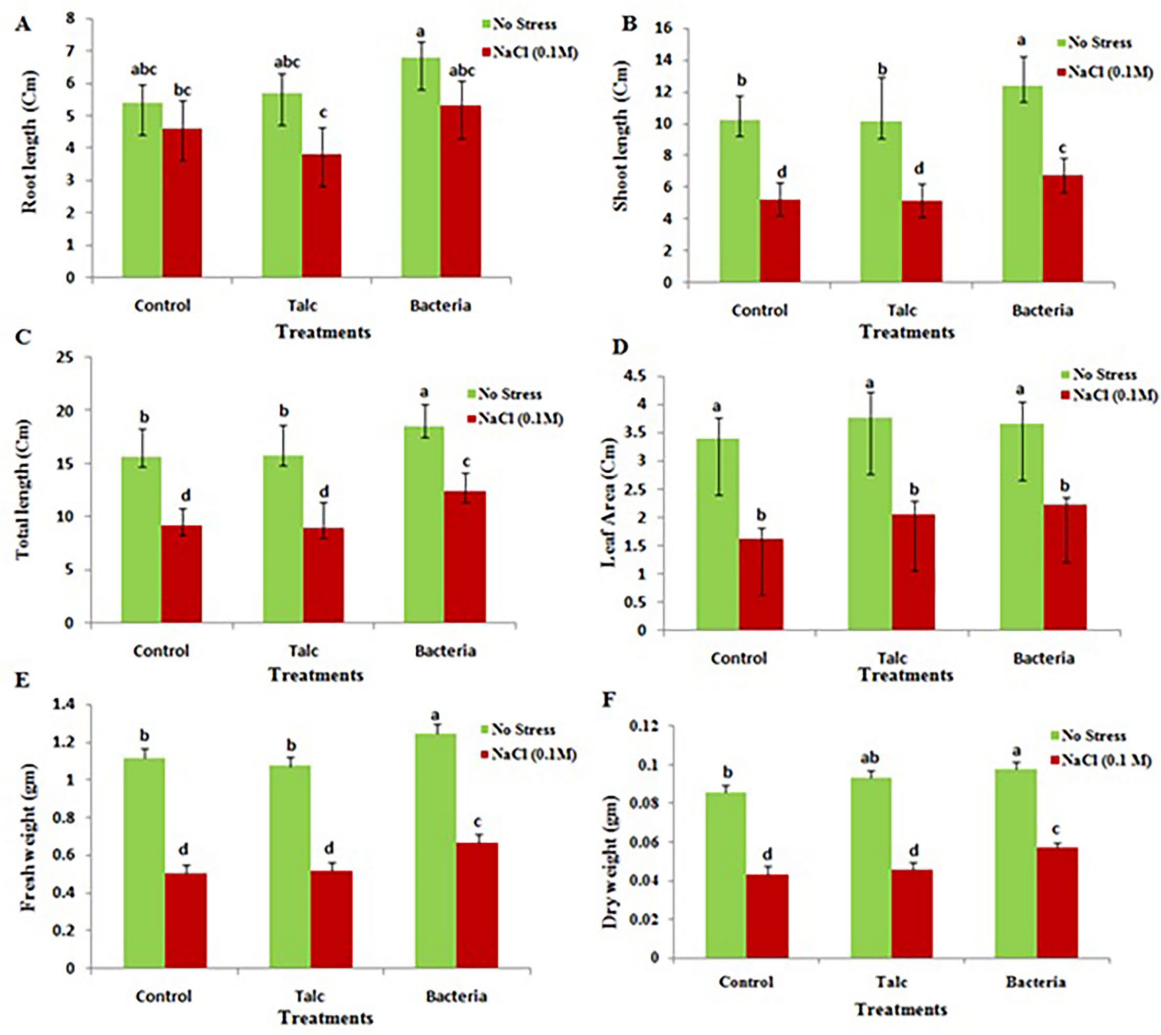
Effect of salinity stress on various growth parameters of differently coated mustard seedlings. **(A)** Root length, **(B)** shoot length, **(C)** total length, **(D)** leaf area, **(E)** fresh weight, and **(F)** dry weight. Data presented are mean ± SE of three replicates. Different letters indicate statistically significant difference between each treatment (DMRT *p* < 0.05).

The results of physiological parameters of treated and non-treated seeds are shown in Figure 3. Plant height along with shoot and root length was recorded maximum in mustard seedlings which were coated with formulation of *Bacillus flexus* both in presence and absence of salt stress. However, there was no significant difference observed for root and shoot length in control and talc coated seedling both under presence or absence of salt stress (Figures 3A–C). The result for leaf area showed no significant difference between condition of presence and absence of salt stress (Figure 3D). However, a significant reduction in leaf area was observed in coated and non-coated seedlings under presence of salt stress. Mustard seedlings coated with *Bacillus flexus* were also recorded best for biomass (fresh weight and dry weight) under both conditions (presence and absence of salt). The control and talc coated seedlings exhibited similar results and did not show any significant difference with each other under both presence and absence of salt stress (Figures 3E,F).

### 3.2 Biochemical properties and antioxidant status of mustard seedlings

#### 3.2.1 Photosynthetic pigments

**Figure 4:**
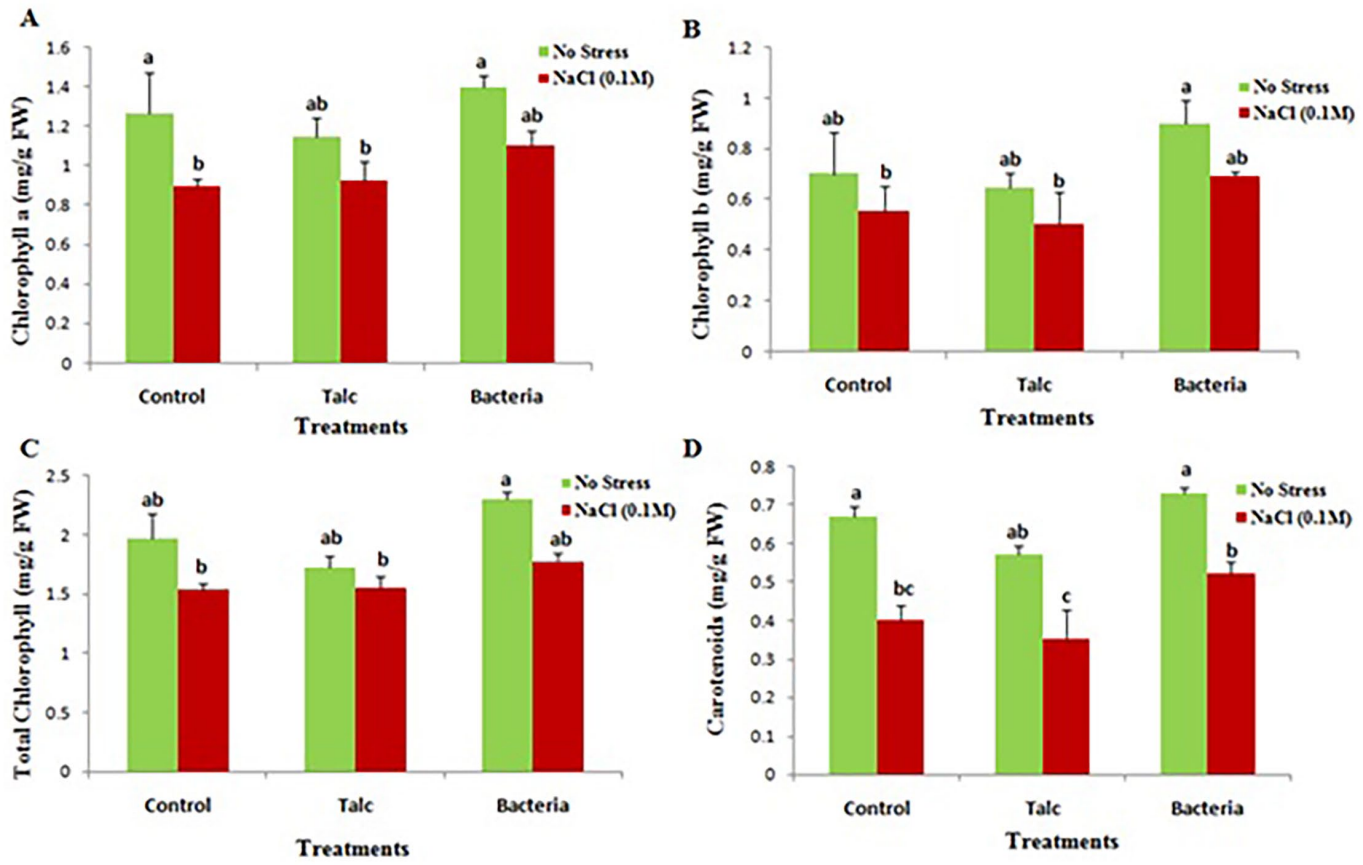
Effect of salinity stress on photosynthetic pigment content of differently coated mustard seedlings **(A)** Chlorophyll a, **(B)** chlorophyll b, **(C)** total chlorophyll, and **(D)** carotenoids. Data presented are mean ± SE of three replicates. Different letters indicate statistically significant difference between each treatment (DMRT *p* < 0.05).

The photosynthetic pigments (chlorophyll a, chlorophyll b, total chlorophyll and carotenoids) were found noticeably reduced in plants subjected to salt stress. However, seedlings with *Bacillus flexus* coating showed higher pigment contents both in presence and absence of salt. Somewhat similar results were observed for control and talc coated seedlings exposed to salt or without any salt exposure (Figure 4).

#### 3.2.2 Electrolyte leakage

**Figure 5:**
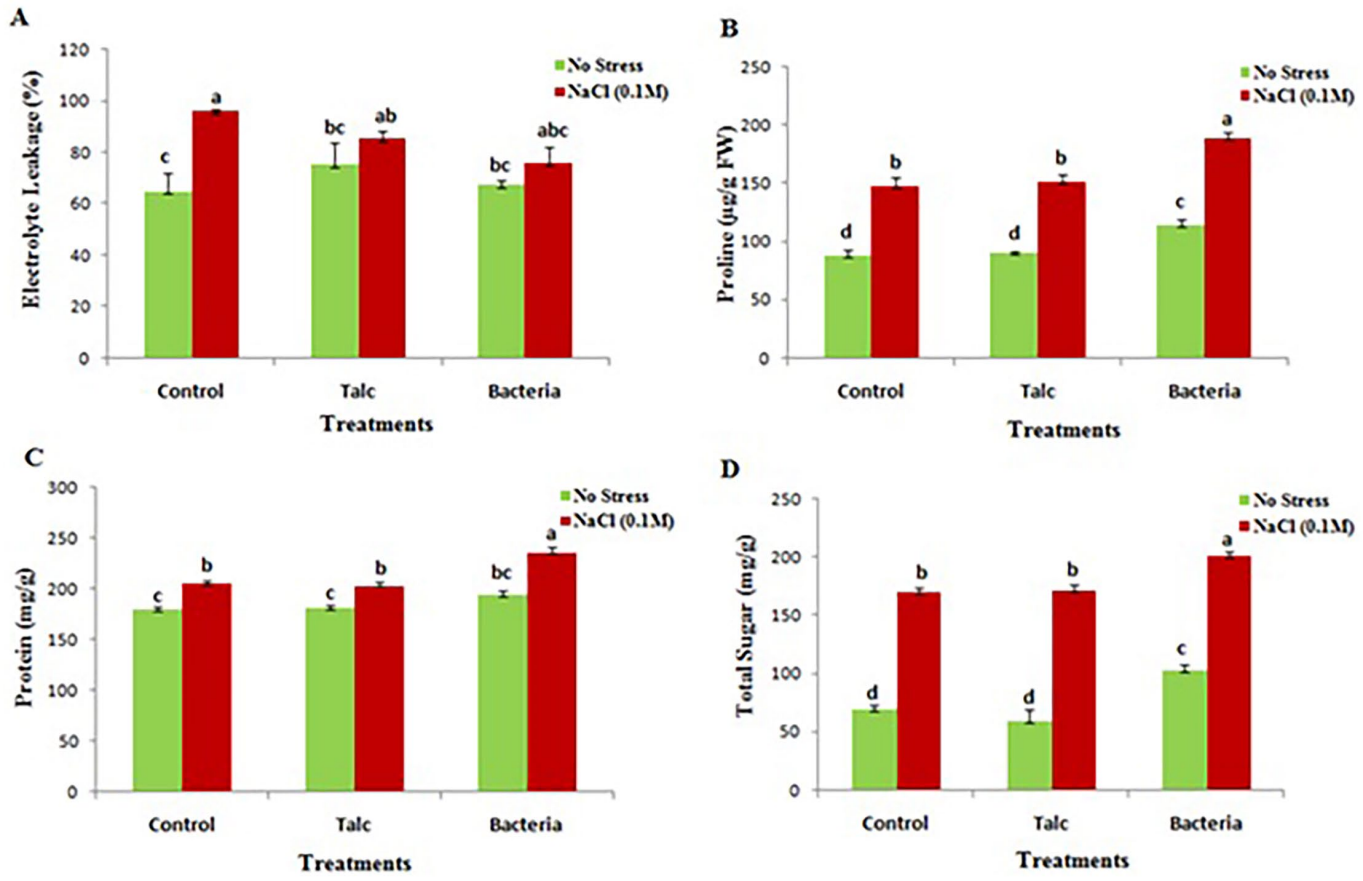
Effect of different seed coating on **(A)** electrolyte leakage, **(B)** proline, **(C)** total soluble protein and **(D)** total soluble sugar content of mustard seedlings under conditions of no stress and with salt stress. Data presented are mean ± SE of three replicates. Different letters indicate significant difference between each treatment (DMRT *p* < 0.05).

The minimum electrolyte leakage (64 ± 7.4%) was recorded in control seedlings which were not subjected to any stress, while the salt treated control seedlings were observed for maximum electrolyte leakage (96 ± 0.72%). In presence of salt the *Bacillus flexus* coated seedlings showed minimum electrolyte leakage (85.4 ± 2.9%) however no significant difference was observed between talc and bacterial coated seedlings in absence of salt stress (Figure 5A).

#### 3.2.3 Proline

The accumulation of proline was elevated in seedlings coated with *Bacillus flexus* under conditions of no stress or with salt stress (113 ± 4.9 μg/g and 188 ± 8.05 μg/g respectively). In case of control and talc coating no significant difference was observed under conditions of no stress or with salt stress (Figure 5B).

#### 3.2.4 Total soluble protein and sugar

Protein and sugar content were also observed high in those seedlings which were coated with *Bacillus flexus* both in presence and absence of salt stress while no significant difference was observed in control and talc coated seedlings when subjected to stress or under condition of no stress (Figures 5C,D).

#### 3.2.5 Malondialdehyde

**Figure 6:**
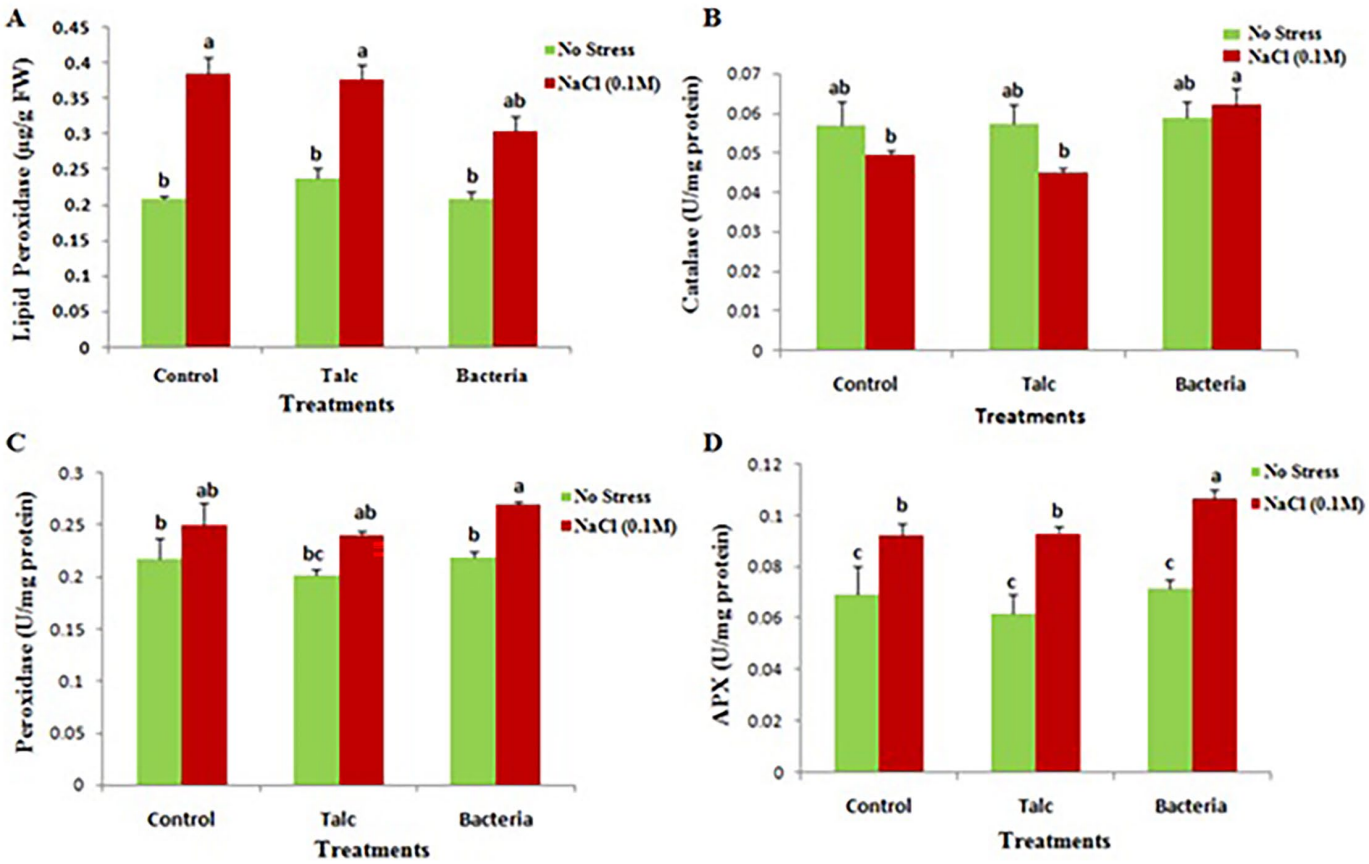
Effect of salinity stress on **(A)** malondialdehyde (MDA), **(B)** catalase, **(C)** peroxidase and **(D)** ascorbate peroxidase activity of differently coated mustard seedlings. Data presented are mean ± SE of three replicates. Different letters indicate significant difference between each treatment (DMRT *p* < 0.05).

The MDA content was found minimum (0.304 μg/g) in *Bacillus flexus* coated seedlings under exposure to salinity stress while in absence of salt stress no significant difference was observed in any treatment (Figure 6A).

#### 3.2.6 Antioxidant enzymatic activity

An elevation in catalase activity was observed (0.058 U mg^−1^ protein) in *Bacillus flexus* coated seedlings under presence of salt stress while in absence of salt stress no difference was observed in any treatment (Figure 6B). The peroxidase and ascorbate peroxidase activity were found maximum (0.27 U mg^−1^ protein and 0.106 U mg^−1^ protein respectively) in *Bacillus flexus* coated seedlings subjected to salt stress. However, no significant difference was recorded in between treatments under absence of salt stress (Figures 6C,D).

## 4 Discussion

Salinity and its generation are a dynamic process and have been considered as a major limiting factor affecting productivity of agricultural soil, plant health and the living organisms within. In this regard, plant growth promoting rhizobacteria has been well known for their contribution in enhancing plant growth as well as for their ability to mitigate the effects of multifarious abiotic stresses including soil salinity in plants (Kumawat et al., 2022; Slimani et al., 2023; AbuQamar et al., 2024; Ansari et al., 2025). The present study intended to evaluate the effectiveness of a salt tolerant rhizobacteria *Bacillus flexus* for enhancing seed germination and growth of mustard seedlings against salinity stress. Mustard plant was chosen for this study because salinity is known to impose a hyper ionic and hyper osmotic stress that interferes with germination, growth and physio-biochemical properties of mustard seedlings (Parti et al., 2003; Ahmad et al., 2015; Arora et al., 2021).

During initial developmental stage, salinity caused noticeable delay and reduction in seed germination as well as seedling growth characteristics. The roots were observed to be affected more adversely as compared to the upper shoot part under conditions of exposed salinity (≥ EC 4.0dS m^−1^, i.e., ~40 mM). Toxic level of Na^+^ and Cl^−^ ions create an outside osmotic potential that avoids water uptake and increased dormancy of seeds under salinity stress (Dimkpa et al., 2009; Nadeem et al., 2014). According to Singh and Panda (2012), the salinity (ECs) from about 2.1 to 3.5 dS m^−1^ of the upper soil profile decreased grain yields in mustard by 12–14% and straw yields by 18–19%. Sarma et al. (2012) reported in their study with mustard that germination time was considerably increased with a rise in salt concentration. Salinity influences germination time more than the germination percentage. This implies that increase in salt concentration results in prolongation of germination time as well as reduce plant growth, seed yield and total lipids of seeds (Parti et al., 2003). In the present study, significant reduction in seed germination, shoot and root length and biomass were found in mustard seedlings when exposed to NaCl induced salt stress, however seed coating with *Bacillus flexus* was observed to minimize the negative impacts of salinity on germination and seedling growth which is in conformity with earlier studies (Arvin et al., 2012; Ahmad et al., 2015).

Photosynthesis is one of the primary plant processes related to its growth and productivity. Slimani et al. (2023) reported disturbance in photosynthetic processes under salinity stress because of accumulation of toxic ions and decrease in water and osmotic potential. Here in the present study, it was observed that the application of NaCl induced salinity stress negatively affected the synthesis of chlorophyll and carotenoids. The reduction in pigment content under salt stress might be due to the impairment in delivery of crucial ions such as Fe^2+^, Mg^2+^, Mn^2+^, and Zn^2+^ that take part in chlorophyll synthesis. Carotenoids possess an excellent antioxidant property and scavenge ROS production thus giving photo protection to chlorophyll. Therefore, decrease in carotenoids content under NaCl stress overpowers the production of ROS that consequently induce oxidative injury to DNA, RNA and proteins thus affecting overall plant growth. The application of *Bacillus flexus* was observed to keep the chlorophyll and carotenoids content to an appreciable level in the present study and the results corroborates with the findings of Ahmad et al. (2015) and Bharti et al. (2016). The reason behind the increased pigment content might be the production of phytohormones that contributes for the stimulation of chlorophyll content and maintain nutrient balance hence the chlorophyll synthesis increases in bacteria inoculated/coated plants (Abbas et al., 2019; Mukhtar et al., 2020; Slimani et al., 2023).

Abiotic stresses are responsible for increased electrolyte leakage because such stresses cause displacement of membrane associated calcium from plasma lemma, thus damaging the membrane permeability consequently leading to higher efflux of electrolytes from plant cell or tissues (Garg and Manchanda, 2009; Kang et al., 2014). The findings of present study showed that *Bacillus flexus* coated mustard seedlings exhibited less electrolyte oozing as compared to non-coated seedlings under conditions of salinity stress. These findings indicated *Bacillus flexus* coating maintained the integrity and stability of cells and tissues of mustard seedlings while non-coated seedlings suffered more due to higher membrane injury leading to higher electrolyte leakage (Garg and Manchanda, 2009).

The increased protein content plays an important role in stress tolerance by preventing from various cellular damages (Campbell and Close, 1997) while soluble sugars are considered as important osmolytes and their increased concentration imparts osmotic adjustment for alleviating salinity stress (Naseem and Bano, 2014). In the present study protein and sugar content were also observed to be high in seedlings coated with *Bacillus flexus.* An elevated accumulation of proline was also observed in seedlings coated with *Bacillus flexus* under absence and presence of salinity stress. An increased concentration of proline in mustard seedlings coated with *Bacillus flexus* might be due to upregulation of proline biosynthesis pathway thus keeping the higher concentration of proline in presence of salinity stress, which further helps in maintaining the cell membranes under salt exposure. Similar results have also been reported by several researchers (Yoshiba et al., 1997; Qurashi and Sabri, 2013; Bharti et al., 2016).

A high level of cellular ROS in the presence of salt stress is responsible for lipid peroxidation of membranes (Bharti et al., 2016; Mukhtar et al., 2020; Ansari et al., 2025). Plants possess various antioxidants enzymes and other antioxidant molecules which help in lowering the ROS level and combating oxidative stress. Results observed in the present study showed a reduction in MDA level in seedlings coated with *Bacillus flexus* under salt stress condition. The present finding is supported with findings of Ahmad et al. (2015), which reported that rhizobacteria improved lipid component of mustard seed under salt stress condition. Under NaCl induced salt stress condition MDA concentration was found to be lowered, however activities of the enzyme’s catalase, peroxidase and ascorbate peroxidase were observed to be increased in seedlings coated with *Bacillus flexus* in seedlings exposed to salinity stress. Catalase an important antioxidant enzyme plays a very essential role in detoxification of ROS under stressful conditions by dismutating hydrogen peroxide directly. POX induces the conversion of hydrogen peroxide into water and oxygen and protects plants from damages induced by salt stress. APX in comparison to CAT and POX enzymes has more affinity for hydrogen peroxide therefore plays an important role in detoxification of ROS under stressful conditions (Khan et al., 2010; Egamberdieva et al., 2019; Mukhtar et al., 2020; Arora et al., 2021; Ansari et al., 2025).

In the present study, the application of *Bacillus flexus* was observed to restore and improve percent germination, biomass, root shoot length, leaf area, osmolytes, chlorophyll and carotenoid contents to an appreciable level. The antioxidant enzyme activity in bacteria coated mustard seedlings was also found best in comparison to control or talc coated seedlings. The results revealed that, under NaCl imposed salinity stress MDA concentration was found to be reduced however catalase, peroxidase and ascorbate peroxidase enzyme activity were enhanced in bacteria coated seedling in comparison to non-coated NaCl stressed seedlings. These findings clearly indicated the protective role of rhizobacteria *Bacillus flexus* for mustard seedlings against adversities imposed by salinity stress.

## 5 Conclusion

Our findings indicates that the selection and use of salt tolerant rhizobacteria possessing multifarious PGP attributes along with osmolyte producing capability could be an effective strategy for the enhancement of plant growth in saline environment. The obtained results related to improved germination, physiological parameters, biochemical properties and antioxidant status of mustard plant treated with rhizobacteria in presence and absence of NaCl clearly indicating the protective responsibility of rhizobacteria *B. flexus* for mustard plant against salinity stress condition. There are very limited studies were reported regarding mustard plant growth promotion by plant growth promoting rhizobacteria under salinity stress condition. Therefore, the results obtained from the present study will definitely contribute in expanding the available information. The use of salt tolerant PGPR might be proved beneficial in prospering strategies to promote plant growth in saline soils because they not only have tolerance to high salinity but also improved crop productivity but much is yet to be explored at biochemical and molecular level that how these PGPR support them as well as associated plant under salinity stress condition. The current finding opens an opportunity to measure the major role of PGPR in reducing the problem of salinity in plants in field conditions.

## Data Availability

The original contributions presented in the study are included in the article/supplementary material, further inquiries can be directed to the corresponding author.
